# Response to supraphysiological testosterone is predicted by a distinct androgen receptor cistrome

**DOI:** 10.1172/jci.insight.157164

**Published:** 2022-05-23

**Authors:** Xintao Qiu, Lisha G. Brown, Jennifer L. Conner, Holly M. Nguyen, Nadia Boufaied, Sarah Abou Alaiwi, Ji-Heui Seo, Talal El Zarif, Connor Bell, Edward O’Connor, Brian Hanratty, Mark Pomerantz, Matthew L. Freedman, Myles Brown, Michael C. Haffner, Peter S. Nelson, Felix Y. Feng, David P. Labbé, Henry W. Long, Eva Corey

**Affiliations:** 1 Center for Functional Cancer Epigenetics, Department of Medical Oncology, Dana-Farber Cancer Institute, Harvard Medical School, Boston, Massachusetts, USA.; 2 Department of Urology, University of Washington, Seattle, Washington, USA.; 3 Cancer Research Program, Research Institute of the McGill University Health Centre, Montréal, Québec, Canada.; 4 Divisions of Human Biology and Clinical Research, Fred Hutchinson Cancer Research Center, Seattle, Washington, USA.; 5 University of California at San Francisco, San Francisco, California, USA.; 6 Department of Surgery, Division of Urology, McGill University, Montréal, Québec, Canada.

**Keywords:** Endocrinology, Oncology, Epigenetics, Prostate cancer, Sex hormones

## Abstract

The androgen receptor (AR) is a master transcription factor that regulates prostate cancer (PC) development and progression. Inhibition of AR signaling by androgen deprivation is the first-line therapy with initial efficacy for advanced and recurrent PC. Paradoxically, supraphysiological levels of testosterone (SPT) also inhibit PC progression. However, as with any therapy, not all patients show a therapeutic benefit, and responses differ widely in magnitude and duration. In this study, we evaluated whether differences in the AR cistrome before treatment can distinguish between SPT-responding (R) and -nonresponding (NR) tumors. We provide the first preclinical evidence to our knowledge that SPT-R tumors exhibit a distinct AR cistrome when compared with SPT-NR tumors, indicating a differential biological role of the AR. We applied an integrated analysis of ChIP-Seq and RNA-Seq to the pretreatment tumors and identified an SPT-R signature that distinguishes R and NR tumors. Because transcriptomes of SPT-treated clinical specimens are not available, we interrogated available castration-resistant PC (CRPC) transcriptomes and showed that the SPT-R signature is associated with improved survival and has the potential to identify patients who would respond to SPT. These findings provide an opportunity to identify the subset of patients with CRPC who would benefit from SPT therapy.

## Graphical abstract

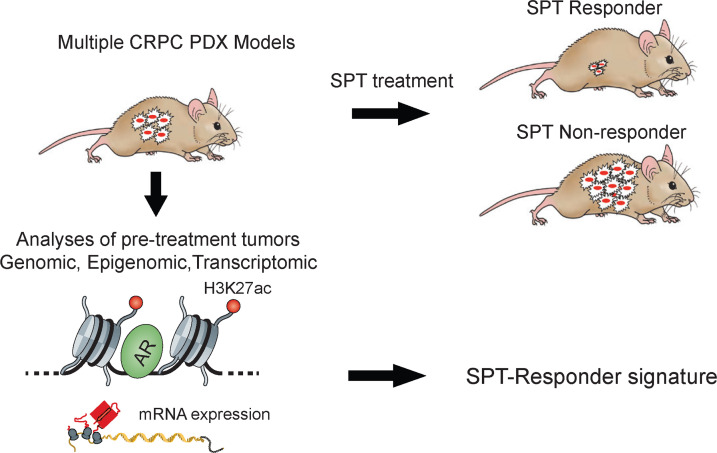

## Introduction

Prostate cancer (PC) is the second most common cancer in men, and it is estimated that 375,000 PC-related deaths occurred worldwide in 2020 (1). Prostate development, as well as PC development and progression, is driven by androgen receptor (AR), a master transcription factor (2). The AR is involved in several transcriptional networks (2, 3), regulating differentiation as well as proliferation. AR transcriptional activity in benign prostate and PC cells is well documented, and inhibition of AR signaling is the primary treatment for recurrent and advanced metastatic PC. In contrast, recent preclinical studies established that activation of AR with supraphysiological levels of testosterone (SPT) can also inhibit late-stage, castration-resistant PC (CRPC) by effectively suppressing DNA damage response (DDR) and proliferation (4–6). In clinical settings, SPT used as bipolar, androgen-based therapy (cycling between castration levels of testosterone and SPT) in patients with metastatic CRPC was safe and effective in a subset of patients with PC, clearly indicating a potential of this therapy for CRPC. However, similar to most other therapies, not all patients benefit from this treatment, and resistance ultimately develops (7–10). Early studies suggested that higher expression of AR and higher prostate-specific antigen (PSA) levels might be associated with better SPT responses**,** and results of a recent analysis of clinical samples indicated a more pronounced response to SPT in patients with mutations in genes associated with DDR (6). However, in another recent clinical study, researchers did not find associations between SPT response and alterations of DDR, *AR,* or *TP53* (11). Clearly, a predictive signature of response would be highly beneficial.

AR binding undergoes significant reprogramming during PC pathogenesis and progression, resulting in gains and losses of AR binding sites (ARBS), and ARBS that are specific to benign prostate, primary PC, and CRPC tumors have been identified (12, 13). The identification of AR cistrome alterations provided a mechanistic understanding of AR signaling–associated events that drive PC progression. In our previous studies, we identified xenograft (PDX) models derived from patients with CRPC that showed significant responses to SPT as well as those that were not affected by SPT treatment (4). The pretreatment-responding (R) tumors exhibited higher AR signaling and a negative association of AR signaling activity with E2F signaling, and proliferation that was not observed in SPT-nonresponding (NR) tumors, suggesting differences in AR signaling and AR regulation of proliferation in R and NR tumors. On the basis of these results, we hypothesized that preexisting differences in AR cistrome and AR-regulated transcriptome in CRPC tumors play an important role in SPT-mediated tumor inhibition and predispose tumor cells to inhibition by SPT. In this study, we sought to determine the epigenomic and transcriptomic differences of AR signaling in R and NR tumors with the objective to identify a signature that will predict response to SPT.

## Results

### SPT-R and SPT-NR tumors exhibit differential AR-binding sites.

**Figure 1:**
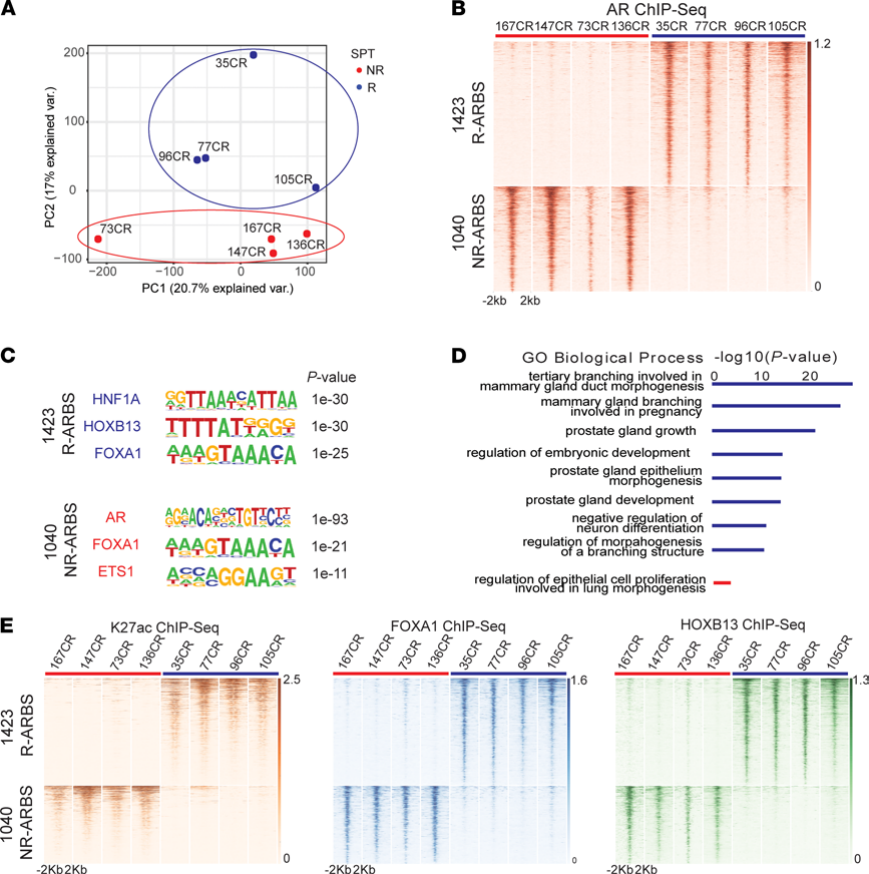
AR ChIP-Seq of pretreatment PDX tumors reveals distinct AR cistromes in SPT responders compared with nonresponders. (**A**) Principal component (PC) analysis plot of AR ChIP-Seq data reveals distinct clustering of R (blue; *n =* 4) and NR (*n =* 4; red) tumors. (**B**) Heat map illustrates differential ARBS identified by comparing R (blue: 35CR, 77CR, 96CR, and 105CR) and NR (red: 167CR, 147CR, 73CR, and 136CR) PDX tumors. A total of 1423 ARBS sites (top) are enriched in R (R-ARBS); 1040 ARBS sites (bottom) are enriched in NR (NR-ARBS; threshold *P*_adj_ ≤ 0.05). (**C**) Significantly enriched motifs in R-ARBS and NR-ARBS. (**D**) GREAT analysis characterizing the biological terms most significantly associated with genes proximal to the R-ARBS (upper; blue) and NR-ARBS (lower; red). (**E**) Heat map indicating H3K27ac, FOXA1, and HOXB13 signal intensity in R (blue: 35CR, 77CR, 96CR, and 105CR) and NR (red: 167CR, 147CR, 73CR, and 136CR) PDX tumors. GO, Gene Ontology; var., variation.

To better understand the biology of SPT effects and identify the epigenomic correlates of R tumors, we set out to investigate differences in AR cistrome between SPT R and NR tumors. AR ChIP-Seq was performed using 8 pretreatment samples: 4 R and 4 NR CRPC PDX models. Responses and characteristics of the PDX models used are shown in Table 1 (4). Unsupervised analysis of AR ChIP-Seq revealed distinct clustering of R and NR tumors (Figure 1A). Supervised analysis of peaks significantly different between R and NR PDX tumors (threshold adjusted *P* [*P*_adj_] ≤ 0.05) identified 1423 ARBS that were highly enriched in R tumors (R-ARBS) and 1040 ARBS that were highly enriched in NR tumors (NR-ARBS; Figure 1B). As expected, the peak annotation assessment revealed that AR binding, including R-ARBS and NR-ARBS, was primarily located at distal intergenic and intronic regions (Supplemental Figure 1B; supplemental material available online with this article; https://doi.org/10.1172/jci.insight.157164DS1).

Next, using motif enrichment analysis, we characterized the unique enhancers identified in the R and NR tumors. For the R-ARBS, we identified HNF1A, FOXA1, and HOXB13 as the most enriched motifs (Figure 1C). HNF1A expression has been associated with a transcriptional program leading to castration resistance (14), indicating a role in the regulation of AR signaling, whereas FOXA1 and HOXB13 are well-known coactivators of AR that are enriched at ARBS (12). Similarly, when cross-referencing R-ARBS with public ChIP-Seq data sets, the most frequent overlap of the R-ARBS was with HOXB13, AR, and FOXA1 binding, with HNF1A also being represented (Supplemental Figure 1C). Although FOXA1 motifs were also enriched in NR-ARBS, enriched NR-ARBS were quite different from the R-ARBS, and they included most prominently AR and ETS1 (Figure 1C). The most frequent overlap in publicly available ChIP-Seq data sets was with AR, HNF4G, and FOXA1 binding (Supplemental Figure 1D). The lack of the AR motif enrichment in R-ARBS and its enrichment in NR-ARBS suggests that SPT supports the oncogenic, canonical AR transcriptional program in NR tumors while fueling an alternate AR program in R tumors.

To further investigate the putative biological functions of the R-ARBS and NR-ARBS, we used the Genomic Regions Enrichment of Annotations Tool (GREAT) (15). The gene ontology biological processes associated with R-ARBS included prostate gland growth, prostate gland epithelium morphogenesis, and prostate gland development (Figure 1D). In contrast, the GREAT analysis of the NR-ARBS identified regulation of cell proliferation involved in lung morphogenesis as the only biological process weakly enriched in NR-ARBS (Figure 1D).

We investigated the H3K27ac status of the R-ARBS and NR-ARBS to determine a potential activity. The results revealed that both were heavily associated with H3K27ac, indicating active enhancers and promoters at their respective sites (Figure 1E). Similarly, R-ARBS and NR-ARBS were both associated with FOXA1 and HOXB13 binding (Figure 1E), which is in agreement with previously published results demonstrating chromatin co-occupancy of AR, FOXA1, and HOXB13 in PC (13).

**Figure 2:**
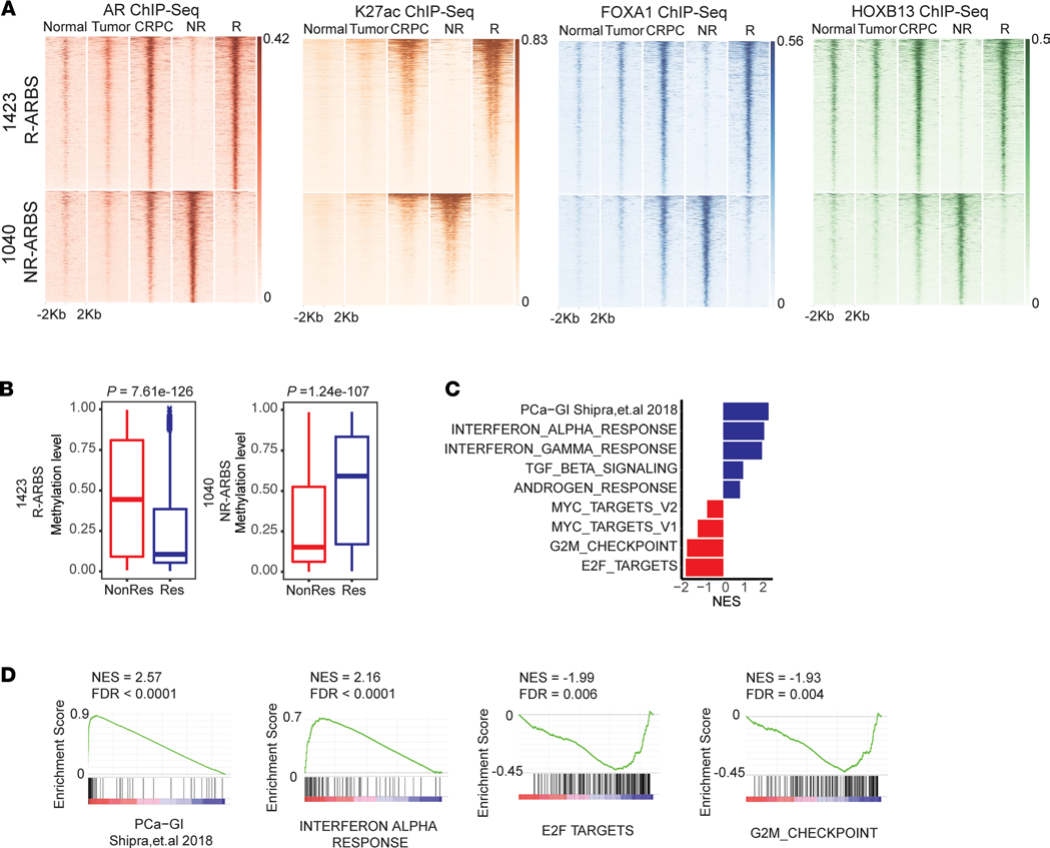
Occupancy of R-ARBS and NR-ARBS during PC progression. (**A**) AR, H3K27ac, FOXA1, and HOXB13 ChIP-Seq signal intensities at R-ARBS and NR-ARBS in benign prostate (Normal: *n =* 13), primary PC (Tumor: *n =* 13), and CRPC tumors (*n =* 15). (**B**) Box plots representing DNA methylation signals across LuCaP R tumors (*n =* 4; blue) and NR tumors (*n =* 4; red) at R-ARBS (left) and NR-ARBS (right). (**C**) Summary of the GSEA results for the hallmark gene sets (blue: enriched in R tumors; red: enriched in NR tumors). (**D**) GSEA enrichment plots for PC-GI, IFN-α response, E2F targets, and G2M checkpoint. NES, normalized enrichment score; Res, responder.

In our previous studies, we showed that the AR cistrome undergoes significant reprogramming during PC progression, resulting in distinct AR-binding patterns across prostate states (12, 13). First, we compared R-ARBS and NR-ARBS occupancy with ARBS in the benign prostatic epithelium and primary PC tumors and detected minimal AR occupancy of R-ARBS and NR-ARBS in both sites, based on the ChIP-Seq signals across these sets of sites (Figure 2A). In agreement with these results, there was minimal overlap between specific normal prostate ARBS and specific primary PC ARBS with either R-ARBS or NR-ARBS (Supplemental Figure 2A). In contrast, there was a strong occupancy of the R-ARBS and NR-ARBS in an unstratified set of metastatic CRPC ARBS (Figure 2A). Moreover, the R-ARBS and NR-ARBS present in CRPC were strongly associated with the H3K27ac of these sites in CRPC and also exhibited stronger FOXA1 and HOXB13 binding in CRPC than in normal prostate or primary PC. Overall, our results show that across the PDX models we have studied, there are clear differences in the AR cistrome of the R and NR tumors, that the R-ARBS and N-RARBS are present in CRPC and not in benign prostate or primary PC, and that the R-ARBS are associated with developmental processes identified with specific AR binding in metastatic CRPC.

The occupancy of enhancers can be reflected by CpG DNA methylation levels (16). Therefore, using EPIC methylation arrays in R and NR PDX tumors, we investigated the methylation at the R-ARBS and NR-ARBS sites. The methylation levels were significantly different and inversely proportional to the AR-binding levels. The R tumors showed relative hypomethylation at R-ARBS compared with NR tumors, and the NR tumors showed relative hypomethylation at NR-ARBS (Figure 2B). Because DNA methylation can be easily detected in cell-free DNA, it might be possible to use the methylation status as an indirect detection method for occupancy at these sites and, therefore, responsiveness to SPT therapy.

### SPT-R and SPT-NR tumors have different transcriptomes and differential AR signaling.

To identify the gene expression profiles associated with SPT responses, we conducted global transcriptome analyses of the 4 R and 4 NR PDX tumors before SPT treatment and identified numerous differentially regulated genes (*n* = 986 genes upregulated in R tumors and 451 upregulated in NR tumors; *P*_adj_ < 0.05, log2 fold change > 1). Gene set enrichment analysis (GSEA) using hallmark gene sets showed weak and not significant enrichment of AR-response genes in the R tumors. Higher output of AR signaling, based on higher serum PSA levels and higher levels of AR transcripts in circulating tumor cells, were suggested to be a potential marker of better SPT response in clinical trials, and our previous study findings also showed higher levels of AR and AR-signaling output in SPT-inhibited PC cell lines and PDXs (4, 6). We hypothesize that these differences might be due to differential methods used to detect AR expression, different patient populations, and differences in biology of AR signaling itself. In support of differential biology, it has been shown that testosterone treatment of AR overexpressing PC-3 cells, but not DU145 cells, resulted in inhibition of proliferation (17., Together, these data suggest that AR expression and expression of AR-response genes alone might not be sufficient to identify SPT responders.

Interestingly, the AR motif was enriched in NR tumors only, indicating that at least some of the genes in the hallmark androgen response gene set are controlled by noncanonical AR signaling. The observation that the HNF1A motif was enriched in R-ARBS led us to evaluate the PC gastrointestinal (GI) signature that was shown to be associated with castration resistance and HNF1A transcriptional activity (14). Indeed, the PC-GI signature was the most highly enriched gene set in the R tumors. The gene sets significantly enriched in NR tumors were MYC targets, G2M checkpoints, and E2F targets (Figure 2, C and D), all associated with regulation of proliferation. Together with the enrichment of the AR motif binding in the NR tumors, these data indicate that in the NR tumors, AR might regulate genes supporting sustained proliferation, leading to resistance to SPT inhibition.

**Figure 3:**
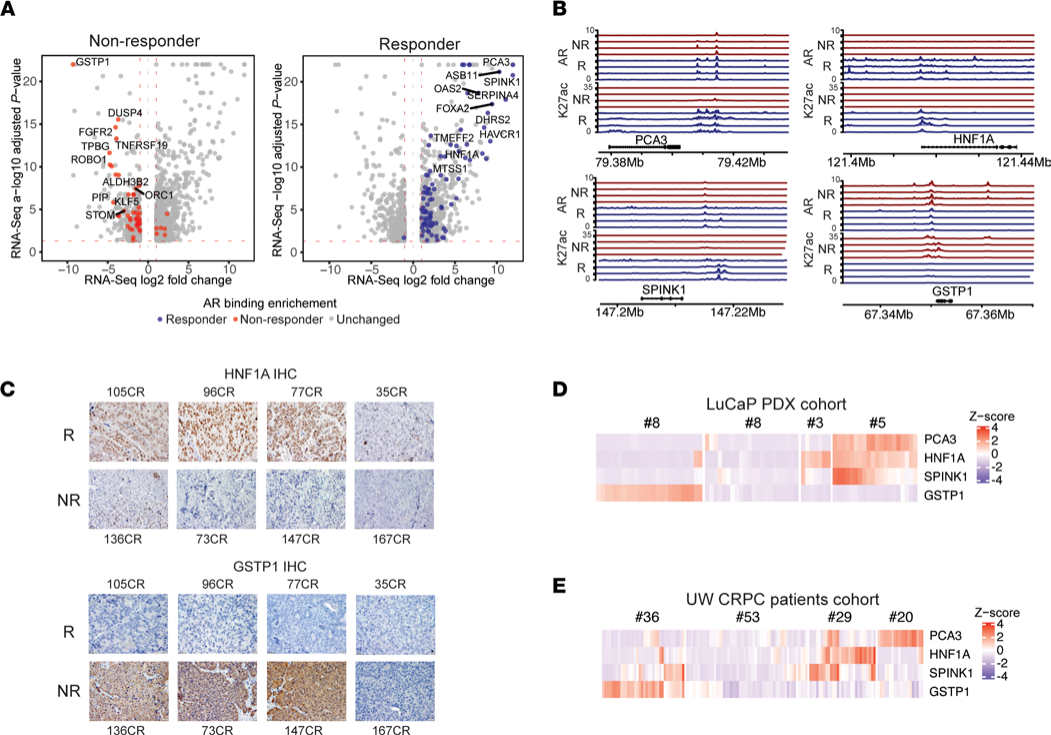
Identification of potential biomarkers of SPT responsiveness by integrating the ChIP-Seq and RNA-Seq data sets from pretreatment PDX tumors. (**A**) Volcano plots of differentially expressed genes between R (*n =* 4) and NR (*n =* 4) PDX tumors. Genes that have nearby R-ARBS are highlighted in blue (right) and those with nearby NR-ARBS are in red (left). (**B**) AR and H3K27ac tracks in regions containing PCA3, HNF1A, SPINK1, and GSTP1. The R tumors (red tracks) show significant AR signal and H3K27ac at the gene promoter of PCA3, HNF1A, and SPINK1, whereas in NR tumors (blue tracks), there is a very little signal of AR and H3K27ac in these regions. In contrast, there is a strong signal of AR and H3K27ac at the GSTP1 region in NR tumors, whereas these bindings are not present in R tumors. (**C**) Representative examples of HNF1A and GSTP1 IHC in R (*n =* 4) and NR tumors. Scale bars: 20 μm. (**D**) Heat map of PCA3, HNF1A, SPINK1, and GSTP1 expression in a cohort of 28 PDX models (*n =* 2–4 tumors per model). (**E**) Heat map of PCA3, HNF1A, SPINK1, and GSTP1 expressions in the UW CRPC cohort (*n =* 138). Numbers above the heat maps indicate the number of tumors.

To evaluate the biological role of the identified differential R-ARBS and NR-ARBS, we interrogated how these differential sites correlate with transcriptional differences, by assigning genes near the R-ARBS and NR-ARBS with a distance cutoff of 50 kb, and we performed cross-analysis with RNA-Seq. This integrated analysis identified genes with higher expression in R tumors with R-ARBS nearby, and genes with higher expression in NR tumors with NR-ARBS nearby. In Figure 3A, the volcano plots are visualizations of the genes differentially expressed between R and NR tumors and genes are highlighted that have differential AR binding sites in their proximity. The most differentially expressed AR-regulated genes (>70-fold) included PCA3, SPINK1, and HNF1A, with high expression in R tumors; and GSTP1 and DUSP4, with high expression in NR tumors (Figure 3A). Importantly, robust AR chromatin occupancy, along with the activation mark H3K27ac, was observed at the most differentially expressed genes (Figure 3B). The differential expression of these genes was consistent across most of the R and NR tumors (Supplemental Figure 2B) and was in good agreement with IHC data (Figure 3C).

We next interrogated the expression of 4 of the most differential AR-regulated, SPT-response associated genes — PCA3, HNF1A, SPINK1, and GSTP1 — in additional PDX tumors. Of 5 additional NR PDX models identified previously (4), 2 did not show generally any expression of GSTP1, but they also did not express high levels of PCA3, and some expressed some levels of the R-associated genes HNF1A, and/or SPINK1. Clustering analysis of the cohort of 28 PDX models showed that there were 9 models that expressed high levels of GSTP1 and generally no PCA3, SPINK1, or HNF1A and 6 models that coexpressed PCA3, SPINK1, and HNF1A and did not express GSPT1. However, this analysis also showed that there were 4 PDX models that of the 3 R-expressed genes mainly expressed HNF1A only, and there were 9 PDX models that expressed very low to no levels of all of these 4 genes of interest (Figure 3D). Interestingly, the latter subset of PDX models included R PDX model LuCaP 35CR and NR-PDX model LuCaP 167CR.

We extended the analysis of the expression of these 4 genes to the University of Washington (UW) cohort with metastatic CRPC, the patient population in which SPT treatment is relevant. The analysis showed that the tumors that expressed PCA3, HNF1A, and/or SPINK1 expressed no or low levels of GSTP1, which is in concordance with PDX analysis separating these 2 populations of tumors. However, the coexpression of the 3 R-associated genes was not as evident in the clinical samples as in the PDX tumors, and there was a cluster of tumors with no to very low expression of all 4 of these genes, as in the PDX cohort (Figure 3E). These results indicate that the expression of each of these genes alone is not a clear indicator of the SPT response, but that does not necessarily negate the possibility of the biological significance of these genes in the SPT response in specific tumors.

### Effects of SPT on the AR cistrome.

**Figure 4:**
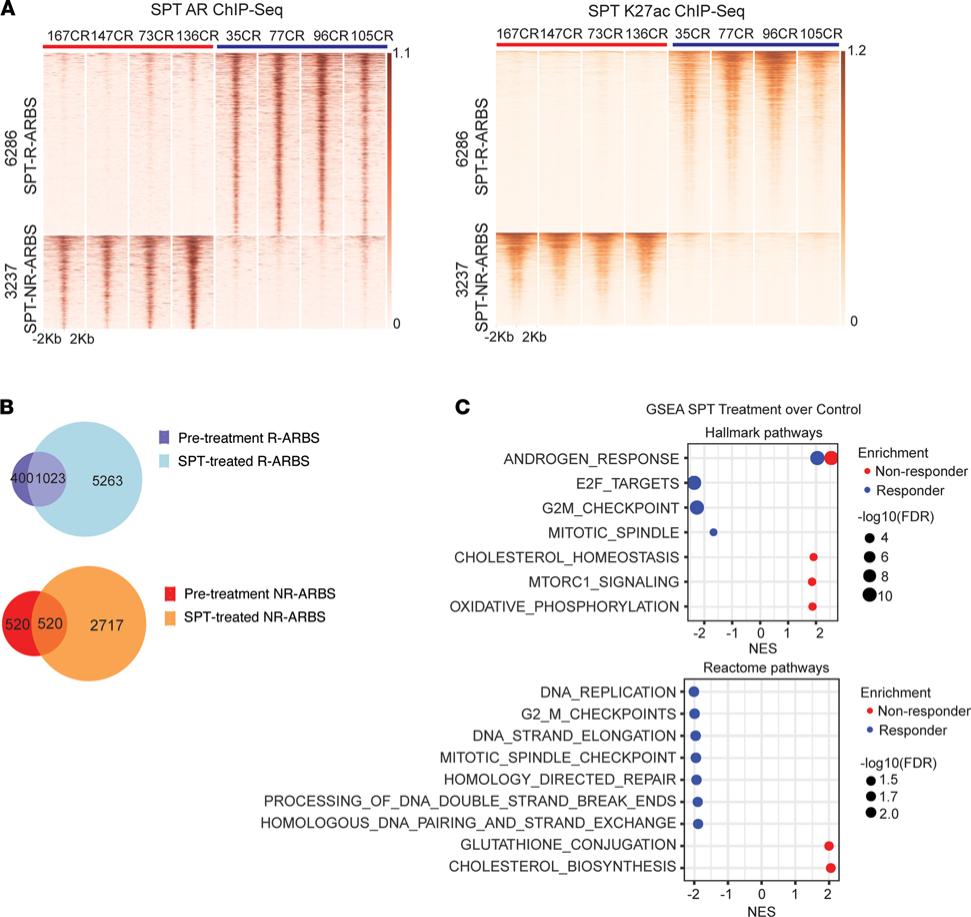
Effects of SPT on AR cistrome and transcriptome. (**A**) Heat maps illustrate differential ARBS (left) and K27Ac signal (right) at the specific ARBS after SPT treatment. Sites were identified by comparing the R (*n =* 4) and NR (*n =* 4) tumors. A total of 6286 ARBS sites (top) are enriched in R tumors (blue: 35CR, 77CR, 96CR, and 105CR; SPT-R-ARBS), and 3237 ARBS sites (bottom) are enriched in NR tumors (red: 167CR, 147CR, 73CR, and 136CR; SPT-NR-ARBS). (**B**) Overlap of ARBS before and after SPT treatment in R (top) and NR (bottom) tumors. (**C**) GSEA of SPT effects in R (blue) and NR (red) tumors. Hallmark gene sets (top), Reactome gene sets (bottom).

We next evaluated SPT effects on the AR cistrome in vivo. We collected tumors from 8 SPT-treated PDX models after 5 days of SPT to ensure we were evaluating the dynamic changes rather than the endpoint tissues of recurring tumors or those containing a higher percentage of necrosis. We performed AR ChiP-Seq analysis of SPT-treated tumors and identified a distinct AR cistrome in SPT-treated R and NR tumors, with 6286 distinct SPT R-ARBS and 3237 distinct SPT NR-ARBS (Figure 4A). The enriched motifs at SPT R-ARBS and SPT NR-ARBS were similar to the unique sites in the pretreatment tumors: the motifs enriched in SPT R-ARBS tumors included FOXA1, HOXB13, and HNF1A; and in SPT-NR-ARBS tumors, AR, FOXA1, and ETS1 (Supplemental Figure 2D). GREAT analysis of SPT R-ARBS showed associations with lateral sprouting of epithelium, steroid hormone–mediated signaling pathways, regulation of morphogenesis and branching structure, and mammary gland development. The only enrichment of biological processes associated with SPT NR-ARBS was labyrinthine layer morphogenesis (Supplemental Figure 2E). These results are similar to what was observed in the pretreatment tumors. Next, we interrogated the concordance of the pretreatment R-ARBS and NR-ARBS with the R- and NR-specific ARBS after SPT treatment and found a significant overlap (*P* < 0.001; Figure 4B). Because some ARBS were lost after SPT treatment, we also performed GREAT analysis of the lost R-ARBS or NR-ARBS. The analysis did not identify any original biological processes associated with these ARBS.

### Effects of SPT on PC transcriptome.

We next analyzed the in vivo effects of SPT in the 4 R and 4 NR PDX tumors using RNA-Seq. In concordance with what we and others have published (reviewed in refs. 4 and 5), the GSEA analysis showed that SPT treatment increased AR signaling (Figure 4C). Notably, the SPT treatment increased AR signaling in both R and NR tumors as determined using the hallmark androgen response gene set, with expression of 36 of 100 genes increased in both R and NR tumors. Yet, 15 additional AR-regulated genes in this set were upregulated in R tumors only, and expression of 17 AR-regulated genes was increased in NR tumors only. These differentially expressed genes represent genes with diverse cellular functions related to the regulation of proliferation and tumor aggressiveness. To further interrogate differences in SPT effects on AR-regulated gene expression in R and NR tumors, we queried in vivo SPT effects on the AR-repressed, AR-induced, biphasic, and inverse biphasic AR-regulated gene sets as identified by Nyquist et al. (18). In concordance with the tumor responses to SPT, SPT resulted in a larger decrease in expression of the AR-biphasic genes enriched for cell cycle and E2F targets and of G2M checkpoint genes in R tumors than in NR tumors (Supplemental Figure 3A). In NR tumors, SPT resulted in a larger decrease in expression of AR-repressed genes that are associated with regulation of eukaryotic translation initiation and elongation, androgen response, and MTORC1 signaling. SPT also resulted in larger increases in expression of AR-induced genes associated with metabolic and biosynthetic programs. Together, these results suggest SPT effects on AR signaling result in differential biological outcomes depending on AR cistrome and transcriptome alterations.

In concordance with published data, the GSEA analysis also showed that SPT treatment decreased E2F targets, G2M checkpoint, and mitotic spindle gene set expression, as well as that of multiple gene sets associated with DNA damage response in R tumors (Figure 4C). Cholesterol homeostasis and biosynthesis, oxidative phosphorylation, and MTORC1 signaling gene sets were enriched in NR tumors. Interestingly, when analyzing SPT-elicited changes in the transcriptome in each R tumor separately, the analysis clearly showed alterations in additional gene sets that were specific to 1 or 2 models only (Supplemental Figure 3B), highlighting the overall heterogeneity of the mechanisms of SPT responses in CRPC leading to tumor inhibition.

### A signature associated with SPT response in CRPC.

**Figure 5:**
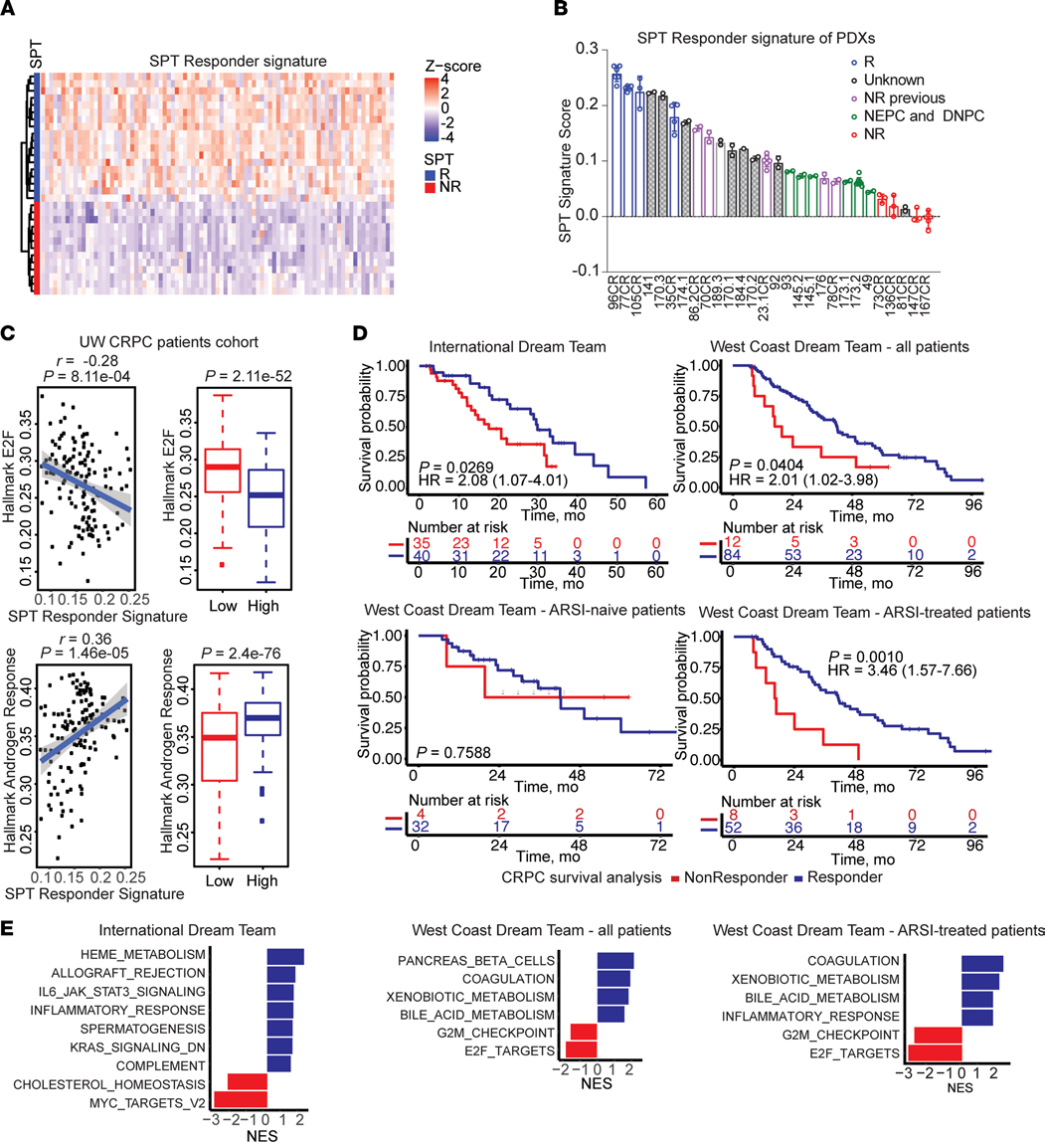
SPT-R signature in PDX models and in patient cohorts. (**A**) Heat map of the SPT-R signature in 4 R and 4 NR PDX models (*n =* 3–6 tumors of different passages per each model). (**B**) Plot of SPT-R signature in the larger LuCaP PDX cohort (*n =* 2–5 different tumors for each PDX model; data reported as mean ± SD). (**C**) Correlation of hallmark E2F signature (top) and hallmark androgen response signature (bottom) with SPT-R signature in the UW CRPC cohort (*n =* 138). The box plots show the groups based on the median of SPT-R signature. (**D**) Kaplan-Meier curves and univariable analysis comparing the survival probability for R (blue) and NR (red) patients in the SU2C-IDT, a discovery cohort (top left), and SU2C-WCDT validation cohort (top right). Univariable analysis of a subset of ARSI-naive patients (bottom row, left) and ARSI-treated patients (bottom row, right). (**E**) GSEA of the hallmark gene sets enrichment (*P*_adj_ < 0.05) in patients determined to be R (blue) or NR (red) in SU2C-IDT (left); SU2C-WCDT, all patients (middle); and SU2C-WCDT ARSI-treated patients (right). DNPC, double-negative prostate cancer; NEPC, neuroendocrine prostate cancer.

We next sought to identify an SPT-R signature on the basis of the analysis of pretreatment control tumors and not the gene alteration by the treatment. Multiple tumors of different passages for each PDX model were included in this analysis. The integrated AR ChIP-Seq and RNA-Seq analyses of the R tumors identified an SPT-R signature of 87 genes (Figure 5A, Table 2). Because the R tumors had higher expression of hallmark AR-response genes and PC-GI signature genes, and low E2F-signaling gene expression, we evaluated overlaps of the SPT-R signature with these gene sets. The SPT-R signature is clearly distinct, because there is minimal overlap of these 3 gene sets (Supplemental Figure 3C).

To determine whether the SPT-R signature can be used as an early indicator of SPT therapy response, we applied this signature to RNA-Seq data from the additional PDX tumors in our original study that measured tumor regression and tumor progression after SPT exposure (4). The SPT-R signature identified all additional NR PDX tumors as NR tumors, because their SPT-R signature score was lower than the scores of the R PDX tumors (Figure 5B). Interrogating RNA-Seq of the additional LuCaP PDX models, we found that LuCaP 141, 174.1, and 189.3 had high SPT-R signatures, suggesting that these PDX tumors would be SPT-R. Importantly, neuroendocrine CRPC LuCaP PDXs (specifically, 49, 93, 145.1, 145.2, 173.1, and 208.2) and double-negative PC (LuCaP 173.2) that do not express AR, and therefore are not susceptible to SPT effects, had low SPT-R signature scores, consistent with the presumption that these are NR tumors.

### Evaluating SPT-R signature in patient cohorts.

To evaluate the clinical relevance of the SPT-R signature, validation in patient samples is needed. Transcriptomes of tumors from patients in SPT clinical trials are not available at present, precluding the examination of the SPT-R signature in cohorts of patients receiving SPT. Therefore, we queried associations of the SPT-R signature with AR- and E2F-signaling activities in UW and Stand Up to Cancer International Dream Team (SU2C-IDT) CRPC transcriptomic data sets (19, 20), because our previous analysis of the PDX tumors showed that R tumors have higher AR signaling and lower expression of E2F-associated genes.

Our analysis showed that the SPT-R signature is positively associated with AR signaling and negatively associated with E2F signaling in both of these CRPC data sets (Figure 5C and Supplemental Figure 3D), highlighting the differential activity of these pathways in clinical specimens based on the SPT-R signature score. In addition, we used data from patients from SU2C-IDT where survival data were available and when the patients were dichotomized using survival cut-point function (Supplemental Figure 3E). The patients with a high SPT-R signature had improved survival compared with the patients with low SPT-R signature (SU2C-IDT: HR = 2.08, *P* = 0.0269; Figure 5D). Next, we used the SU2C West Coast Dream Team (SU2C-WCDT) patients (21, 22) as a validation cohort. Our results confirmed an improved survival of patients with the higher SPT-Responder signature in this cohort (HR = 2.01; *P* = 0.0404; Figure 5D) (21, 22). Interestingly, when we stratified patients in SU2C-WCDT according to whether they received secondary AR-signaling inhibitors (ARSIs), the ARSI-treated patients with a high SPT-R signature had an improved survival (HR = 3.46; *P* = 0.0010), whereas the SPT-R signature in ARSI-naive patients was not associated with survival benefits (*P* = 0.9669; Figure 5D).

These results indicate that the SPT-R signature is associated with differential AR signaling and response to ARSIs in clinical specimens. Furthermore, in concordance with our previous results showing that SPT-R tumors have lower expression of E2F targets and G2M checkpoint genes, or MYC targets, the patient tumors identified as potential responders on the basis of the SPT-R signature also exhibited a lower expression of genes in these data sets (Figure 5E).

## Discussion

SPT therapy is a well-tolerated treatment that has demonstrated antitumor efficacy in a subpopulation of patients with CRPC. Therefore, identification of signatures predicting SPT response would have important implications for patient treatment selection. Using CRPC PDX tumors, we provide the first preclinical evidence, to our knowledge, that SPT-R tumors exhibit a distinct AR cistrome when compared with SPT-NR tumors, indicating the differential biological role of AR signaling in R and NR tumors. Integrative analysis of the AR ChIP-Seq and RNA-Seq of the pretreatment tumors led to the identification of an SPT-R signature that differentiates between SPT-R and SPT-NR tumors in a preclinical setting. Because transcriptomes of SPT-treated clinical specimens are not available, we interrogated available CRPC transcriptomes and showed that the SPT-R signature is associated with improved survival. These findings, together, have a clear clinical translatability, providing an opportunity to identify a subset of patients with CRPC who would respond to SPT with improved survival.

AR is a prostate development and PC progression master regulator, and it is well accepted that during PC progression, AR-signaling alterations take place. Our results showed that R-ARBS are generally not present in normal prostate or primary PC but are selectively present in CRPC. In addition, the R-ARBS, as well as CRPC-specific ARBS, are located in the proximity of genes associated with developmental processes, reviving the lineage-specific programs during CRPC progression. Given that the SPT therapy is used in the CRPC setting, where it exhibits its tumor inhibitory effects, our findings support the hypothesis that the R-ARBS are biologically and clinically relevant, and provide a rationale for SPT use in patients with CRPC harboring R-ARBS cistrome.

Our objective was to investigate common characteristics of the SPT responders to identify biomarkers or signatures that would identify R tumors with consideration of the heterogeneity of advanced CRPC and responses to SPT. Although we identified multiple AR-regulated genes associated with SPT responses in pretreatment tumors, no single gene can be associated with tumor-promoting or suppressing effects or SPT effects in all PDX tumors or clinical specimens. In our testing PDX cohort, PCA3, HNF1A, and SPINK1 were coexpressed in R tumors. However, the analysis of additional PDX tumors and patient specimens showed there were tumors expressing only 1 or 2 of these 3 genes, suggesting that their roles in biology are independent, and expression of these 3 factors separately would not identify SPT responders with high precision. Interestingly, GSPT1, a gene epigenetically silenced in a large fraction of localized PC, was expressed highly in the 4 NR tumors, and its expression was generally mutually exclusive with these 3 genes in the extended PDX cohort as well as in the clinical specimens, suggesting a potential biological role of GSPT1 in resistance to SPT therapy.

SPT exerts its effects via AR signaling and alterations of PSA, an AR-regulated gene, have been used to monitor SPT effects in preclinical and clinical settings. It is important to note that in our studies, we showed that SPT treatment elicited upregulation of hallmark androgen response genes in both R and NR tumors. However, although some genes in the hallmark AR response gene set were upregulated in both R and NR tumors, there was also a subset of these genes specifically increased in R tumor only and a subset increased in NR tumors only. These results further demonstrate the uncoupling of AR regulation of proliferation and differentiation, and the differential AR signaling in SPT R and NR tumors. Importantly, these results indicate that predicting SPT response on the basis of alteration of the current androgen response genes set does not appropriately identify R and NR tumors.

DDR alterations in tumors were identified as one of the important aspects associated with SPT tumor inhibition in a preclinical setting (4, 6). Moreover, in clinical settings, patients with mutations in homologous recombination had more pronounced responses to SPT, suggesting that tumors of patients with alterations of expression of genes involved in homologous recombination will more likely respond to the SPT therapy (6). In the present study, we could not draw a similar conclusion about DDR or other genomic alterations associations that were suggested to potentially play a role in SPT responsiveness (e.g., AR*, TP53,* and *RB1*), because our study is limited by a small number of models. However, importantly, the results we report here show that multiple SPT-altered pathways in R tumors are distinct among the 4 R PDX tumors, indicating that different mechanisms of action are involved in transducing SPT-inhibitory effects and that SPT impact on the tumor is dependent on cellular context. For example, DDR-associated gene sets were altered only in LuCaP 96CR and LuCaP 77CR, and not in the other 2 R tumors; and LuCaP 105CR, an R tumor, exhibited significantly different transcriptomic alterations than did the other 3 R tumors. Similar to these results, a minimal overlap in gene expression alterations was associated with enzalutamide resistance when 4 different PC cell lines were used (23). Together, these findings indicate the overall heterogeneity of the mechanisms of responses to AR-targeted treatments in CRPC leading to tumor inhibition and, therefore, limitations to create a reliable response signature based on treatment-induced transcriptomic alterations.

On the bases of the heterogeneity of SPT-induced transcriptome alterations and the finding that the majority of distinct R-ARBS were concordant before and after the SPT treatment, we postulated that a response signature based on the pretreatment differences between R and NR tumors would be the most clinically relevant to the outcome of the SPT treatment and that a signature derived from the enriched R-ARBS and upregulated gene expression in R tumors will predict the SPT response, providing a unique opportunity to identify responders from nonresponders. To our knowledge, this is the first report focusing on differences in AR signaling in pretreatment tumors that subsequently were classified as SPT R and NR tumors in vivo. Such determinations are unique to preclinical models, where replicates of phenotypically and genotypically identical tumors can be evaluated. The diverse response phenotypes associated with SPT tumor inhibition highlighted the need for a signature, and we proposed that the differences in AR signaling in the parental pretreatment tumors are highly clinically relevant to outcomes of the SPT treatments.

We chose to create an SPT-R signature because of the limited association of NR-ARBS with biological processes, and we created an SPT-R signature of 87 genes on the basis of R-ARBS and high expression in pretreatment R tumors. This signature is unique and does not show overlap with the hallmark androgen response gene set despite being generated using genes with AR binding within 50 kb of the TSS. We explain this observation by the differences in how these signatures were derived. The SPT response signature is based on the high expression of genes near the R-ARBS in the pretreatment tumors (not under stimulation of androgen), whereas the hallmark androgen response gene set is based on genes upregulated in LNCaP cells in response to synthetic androgen R1881 in vitro (24).

Our validation of the SPT-R signature in an extended PDX cohort correctly identified 4 additional NR tumors, and all NEPCs and double-negative PC PDX models that do not express AR had low SPT-R signature scores, indicating an SPT NR tumor. However, our SPT response signature identified correctly only 3 of 5 cell lines (18), 2 of 4 SPT-R PC cell lines, and 1 SPT-NR cell line. We hypothesize that this discrepancy is related to the differences between in vivo and in vitro conditions, because our analysis showed that there is very low expression of multiple genes of the SPT-R signature in cell lines grown in vitro. Only 22% of the genes in the SPT-R signature were expressed in cell lines at fragments per kilobase of exon per million mapped fragments (FPKM) ≥ 5. In contrast, using the same criteria for the patient cohorts, the percentages of genes expressed were 66% in IDT SU2C and 83% genes WSDT SU2C.

To investigate the SPT-R signature in a clinical context of CRPC, we used the transcriptomic data sets of UW and SU2C CRPC cohorts, because the transcriptomes of tumors from patients who underwent SPT therapy are not yet available. It was previously suggested that low E2F and high AR signaling in tumors might be an indication of positive SPT response (18). Our analyses showed that the SPT-R signature is negatively associated with E2F signaling and positively associated with AR signaling in both of these cohorts. However, the overlap of genes in the SPT-Response signature and hallmark androgen response and hallmark E2F signaling is minimal, indicating that the SPT response signature identifies different population of patients than those 2 currently used gene expression signatures. Interestingly, the SPT-R signature was associated with improved survival in 2 cohorts of patients with CRPC and even more so in CRPC tumors from patients who were treated with ARSIs (21, 22). We hypothesize that because the SPT-R signature is based on differential AR signaling, the identified AR signaling differences can also play a role in responses to ARSIs.

### Conclusions.

In summary, our results demonstrate that SPT responders and SPT nonresponders have differential AR cistromes. Using integrative analysis of ChIP-Seq and RNA-Seq of pretreatment R tumors, we identified an SPT-R signature that can categorize SPT-R and SPT-NR tumors in preclinical settings. In clinical settings, the SPT-R signature was associated with improved survival of patients with CRPC. The validation of the SPT-R signature in patients treated with SPT is needed to further support the clinical relevance of the signature.

## Methods

### Study design

The overall objective of this study was to identify differences in the AR cistrome of SPT-R and SPT-NR PDX tumors and to identify an SPT-R signature. We used 8 CRPC PDX models: 4 R and 4 NR PDX tumors collected from control and SPT-treated animals for investigation of the AR cistrome, effects of SPT, and SPT-R signature identification. For the SPT-R signature evaluation, we used a cohort of 28 CRPC LuCaP PDXs and transcriptomic data from UW and SU2C cohorts.

### SPT effect in vivo

CB17 male SCID mice (Charles River) were castrated and, 2 weeks after castration, were implanted with tumor bits of LuCaP 73CR, 136CR, 147CR, 167CR, 35CR, 77CR, 105CR, and 96CR. When tumors exceeded 100 mm^3^, animals were randomized to control and treatment groups (*n* = 3–15 per group). Testosterone cypionate (Roadrunner Pharmacy) was administered i.m. every 2 weeks (10 μL of 100 mg/mL); control animals received an injection of 10 μL of sesame oil. Tumor volume and BWs were monitored twice weekly. For ChIP-Seq and RNA-Seq analyses, 1 tumor per group for each model was harvested 5 days after the beginning of the treatment. The remaining animals were sacrificed when tumors exceeded 1000 mm^3^ or if they were becoming compromised. Treatment effects were analyzed using GraphPad Prism 9 software.

### ChIP-Seq analysis

Pretreatment and SPT-treated s.c. PDX tumors (*n* = 1 per group and per PDX model) were processed as described in ref. 13, with Abs to AR (N-20, Santa Cruz Biotechnology), HOXB13 (H-80, Santa Cruz Biotechnology), FOXA1 (ab23738, Abcam), and H3K27ac (C15410196, Diagenode). Libraries were sequenced using 75 bp reads on the Illumina platform at the Dana-Farber Cancer Institute Center for Functional Cancer Epigenetics (CFCE).

#### Peak calling and data analysis.

All samples were processed through the computational pipeline developed at the Dana-Farber Cancer Institute CFCE, using primarily open-source programs. Sequence tags were aligned with Burrows-Wheeler Aligner to build hg19, and uniquely mapped, nonredundant reads were retained (25). These reads were used to generate binding sites with Model-Based Analysis of ChIP-Seq 2 (MACS version 2.1.1.20160309), with a *q* (FDR) threshold of 0.01 (26). We evaluated multiple quality-control criteria on the basis of alignment information and peak quality: (a) sequence quality score; (b) uniquely mappable reads (i.e., reads that can only map to 1 location in the genome); (c) uniquely mappable locations (i.e., locations that can only be mapped by at least 1 read); (d) peak overlap with Velcro regions, a comprehensive set of locations (also called consensus signal artifact regions) in the genome that have anomalous, unstructured, high signal or read counts in next-generation sequencing experiments independent of cell line and of type of experiment; (e) number of total peaks (the minimum required was 1000); (f) high-confidence peaks (i.e., the number of peaks that are 10-fold enriched over background); (g) percentage overlap with known DHS sites derived from the ENCODE Project (the minimum required to meet the threshold was 80%); and (h) peak conservation (a measure of sequence similarity across species based on the hypothesis that conserved sequences are more likely to be functional).

#### Differential binding analyses.

Peaks from each group were used for motif analysis by the motif search findMotifsGenome.pl in HOMER (version 3.0.0), with cutoff value of *q* ≤ 1 × 10^–10^.

#### Sample–sample correlation and differential peaks analysis.

Sample–sample correlation and differential peaks analysis were performed by the Containerized Bioinformatics Workflow for Reproducible ChIP/ATAC-Seq Analysis pipeline (27). Peaks from all samples were merged to create a union set of sites for each transcription factor and histone mark. Read densities were calculated for each peak for each sample and used for the comparison of cistromes across samples. Sample similarity was determined by hierarchical clustering using the Spearman’s correlation between samples. Tissue-specific peaks were identified by DEseq2 (28) with *P*_adj_ ≤ 0.05. A total number of reads in each sample was applied to the size factor in DEseq2, which can normalize the sequencing depth between samples.

#### ChIP-Seq profiles.

Given the varying alignment of reads or fragments across samples, coverage track BigWig files were calculated for each sample that reflected the coverage signal and sequencing depth using the Chilin pipeline (29). The deepTools, version 2.3.5, package *computeMatrix* further computed the average score for each of the samples. Finally, a profile heat map was created on the basis of the scores at genomic positions within 2 kb upstream and downstream of the ARBS. All samples were ranked by the average score. ChIP-Seq enrichment for transcription factors and histone marks at the loci of selected genes were visualized and plotted using the *karyoploteR* (version 1.12.4) R package (30).

#### Overlap analysis.

The overlap of R-ARBS and NR-ARBS pretreatment and after SPT treatment was tested using Fisher’s exact test in the *GeneOverlap* (version 1.30.0) R package (31).

### DNA methylation

DNA was extracted from pretreatment s.c. PDX tumors (*n* = 1 per model) using the Qiagen All Prep Kit according to the manufacturer’s instructions. Genome-scale methylation analyses of PDX tumor DNA were carried out using Infinium MethylationEPIC BeadChip arrays (Illumina) as described previously (32). Raw data were analyzed in the *minfi* package in R (33), and samples were normalized using the subset-quantile within array normalization method (33, 34). Probes with a detection *P* > 0.01 in 50% or more samples and probes that contained an SNP at the CpG interrogation site or at the single nucleotide extension were removed. The *GenomicRanges* package (35) was applied to intersect the resulting β values with loci of AR binding sites defined by ChIP-Seq calls, and the distribution of β values for different AR binding site groups was computed.

### RNA-Seq

RNA was extracted from s.c., control, pretreatment PDX tumors using STAT-60 (Fisher Scientific, catalog NC9489785), and RNA-Seq was performed as we previously described (4, 36). RNA from the control and SPT-treated PDX tumors for analysis of SPT effects was sent to Novogene for RNA library preparation and mRNA sequencing (Illumina HiSeq Platform, PE150). For RNA-Seq analysis of the pretreatment PDX tumors, we combined the original LuCaP PDX RNA-Seq data set with the new pretreatment PDX tumor RNA-Seq data set (*n* = 3–6 per model). Read alignment, quality control, and data analysis were performed using Visualization Pipeline for RNA-Seq Analysis (VIPER) (37), RNA-Seq reads were mapped by Spliced Transcripts Alignment to a Reference (STAR) (38), and read counts for each gene were generated by Cufflinks (39). Differential gene expression analyses were performed on absolute gene counts for RNA-Seq data and raw read counts for transcriptomic profiling data using DESeq2 (28). Transcriptional signature scores were computed for every sample based on a nonparametric, rank-based method implemented in the *singscore* (version 1.6.0) R package (40). GSEA was done using preranked analysis (GSEA Java, version 4.1.0, ref. 41) with hallmark and Reactome gene sets.

### Immunohistochemical analysis

Sections of paraffin-embedded tissues were stained using HNF1-α Ab (ab272692, Abcam) using antigen retrieval at pH 9, or GSPT1 Ab (sc-66000, Santa Cruz Biotechnology) using antigen retrieval at pH 6 and our standard procedure with ABC kit detection (4).

### SPT-R signature generation

To identify the SPT-R signature, we first selected genes upregulated in R tumors with DEseq2 and adjusted *P*adj ≤ 0.05, and log2 fold change ≥ 1. Genes expressed in only 1 model (FPKM > 5) were removed from the analysis. Next, we identified upregulated genes that also have enriched R-ARBS (*P*adj ≤ 0.05) within 50 kb of the TSS. This resulted in a total of 87 genes being selected as the unidirectional SPT-R signature.

### Analysis of cohort of patients with CRPC

#### Clustering analysis.

*Z*-score normalization was applied to the FPKM value of PCA3, HNF1A, SPINK1, and GSTP1. K-means clustering was applied to the *Z*-score normalized matrix. Heat map visualization of the *Z*-score was obtained using the *ComplexHeatmap* (version 2.2.0) R package (42).

#### Correlation analysis.

Transcriptional signature scores were computed using the *singscore* (version 1.6.0) R package (40) with the SPT-R signature, hallmark E2F, and androgen response gene sets as described previously (43). Correlations were calculated based on the Pearson correlation. A fitted linear regression model with 95% CIs was plotted on the singscore scatter plot. Wilcoxon rank-sum test was performed to compare the 2 groups separated by the median of the SPT-R signature signscore.

#### Survival analysis.

For the survival analysis, the published SU2C-IDT metastatic CRPC data set was used (19). To calculate transcriptional signature scores, read counts were normalized to sequencing depth and TPM transformed. Transcriptional signature scores were computed in the *singscore* (version 1.6.0) R package (40) using a unidirectional SPT-R signature comprising 87 genes. Patients were assigned to the SPT-R or SPT-NR groups according to the cut-off point estimated by the maximally selected rank statistic in the R *maxstat* package (version 0.7–25) (44). Survival analysis was conducted using the *survival* (version 3.2–3) R package (45), Kaplan-Meier curves were calculated and plotted using *survminer* (version 0.4.8) R package (46), and log-rank test was used to evaluate the overall statistical significance as well as the comparison between groups. Benjamini-Hochberg correction was used to correct for multiple testing. SU2C-WCDT patients (21, 22) comprised a validation cohort.

### Data availability

The ChIP-Seq and RNA-Seq data generated in this study were deposited in the NCBI’s Gene Expression Omnibus database (GEO GSE188176).

### Statistics

Statistical analyses were conducted in the statistical computing environment R (version 3.6.0). Statistical comparison for differential peaks was done using DESeq2 (28), which fits negative binomial generalized linear models for each peak and uses the Wald test for significance testing with Benjamini-Hochberg correction for multiple tests. Motif enrichment was calculated using Hypergeometric Optimization of Motif Enrichment (HOMER; version 3.0.0) based on the cumulative hypergeometric distributions. The hypergeometric test of genes provided an accurate annotation enrichment for genomic regions in the GREAT (15) pathway analysis. *P* values were calculated using Student’s *t* test for box plot, and Pearson’s correlation test was used to evaluate the statistical significance of correlations. For Kaplan-Meier survival analysis, the log-rank (Mantel-Cox) test was used to determine significance. *P* < 0.05 was considered significant.

### Study approval

Collection of tumors for PDX establishment was approved by the UW Human Subjects Division IRB (IRB 2341) and after receiving the written consent of the patients. Animal studies were approved by the UW IACUC (protocol 3202-01) and performed in accordance with the NIH guidelines.

## Author contributions

XQ, HWL, and EC designed the study. XQ, MP, MLF, NB, DPL, MCH, MB, HWL, and EC designed the study methodology. JHS, TEZ, CB, EO, SAA, MMP, MLF, HMN, JLC, LGB, and BH contributed to data collection. Resources were provided by PSN, FYF, MCH, and EC. Animal work, PDX sample collection, and IHC were performed by HMN, JLC, and LGB. XQ, DPL, NB, MCH, and BH contributed to bioinformatics and statistical analyses. XQ, MCH, DPL, HWL, and EC contributed to data interpretation and presentation. XQ, HWL, and EC prepared the manuscript. All authors critically reviewed the manuscript.

## Supplementary Material

Supplemental data

## Figures and Tables

**Table 1:**
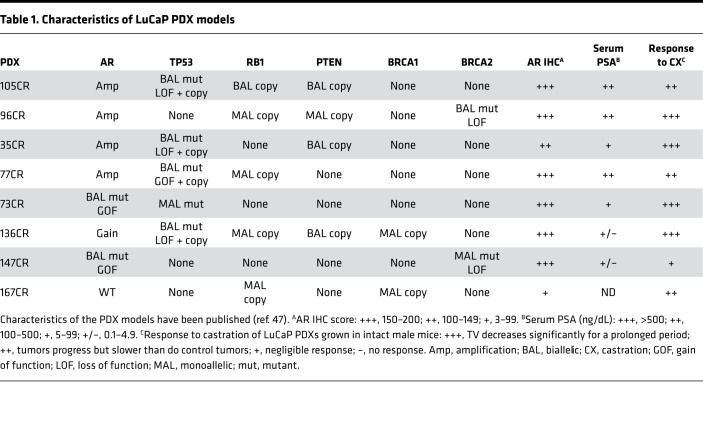
Characteristics of LuCaP PDX models

**Table 2:**
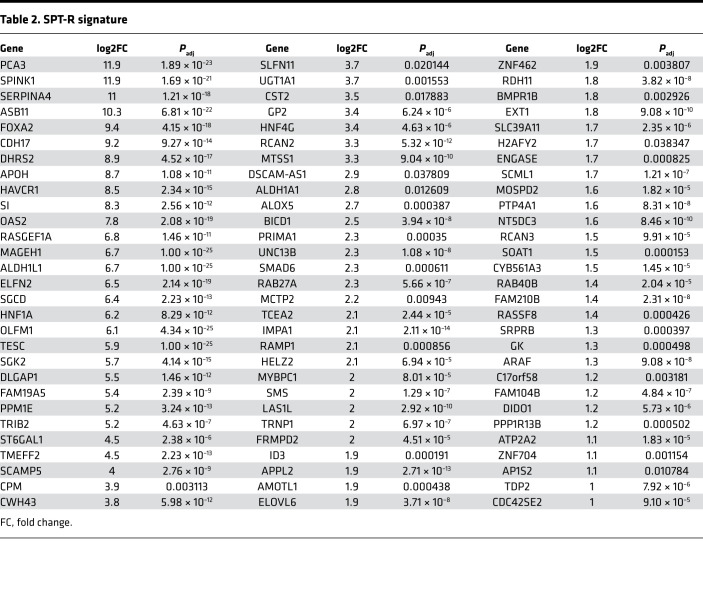
SPT-R signature

## Version 1 05/23/2022

Electronic publication
