# Supplementary material for: Response to supraphysiological testosterone is predicted by a distinct androgen receptor cistrome
Source: JCI Insight. 2022 May 23;7(10):e157164. doi: 10.1172/jci.insight.157164 (PMC9220831; doi:10.1172/jci.insight.157164)
Supplement: Supplemental data [file jciinsight-7-157164-s074.pdf]

Supplemental Figure 1. (**A**) LuCaP PDX tumor responses to SPT treatment. (n=3-15 per group (blue: R, top; red: NR, bottom, mean ± SEM). (**B**) Localization of AR binding sites in R and NR PDX tumors. (**C**) Results illustrating the similarity of publicly available ChIP-Seq datasets ranked by GIGGLE score to R-ARBS and (**D**) in NR-ARBS. The color indicates the biological source of the ChIP-Seq experiments.

Supplemental Figure 2. (**A**) Overlap between NARBS and TARBS with R-ARBS (left) and NR-ARBS (right). (**B**) Gene expression (Log2 FPKM) of PCA3, HNF1A, SPINK1, and GSTP1 in eight PDX model. (blue: R; red: NR, n=2-4 different tumors per each PDX model, mean ±SEM). (**C**) Significantly enriched motifs in SPT-R-ARBS (blue) and SPT-NR-ARBS (red). (**D**) GREAT analysis characterizing the GO biological processes most significantly associated with genes proximal to the SPT-R-ARBS (upper, blue) and NR-ARBS (lower, red).

Supplemental Figure 3. (**A**) GSEA analysis of AR-regulated genes by SPT treatment in R (blue) and NR (red) PDX tumors. (**B**) GSEA analyses of SPT effects in each of four R PDX tumors: Hallmark gene sets (top) and Reactome gene sets (bottom) red: up-regulated, blue: down-regulated. (**C**) Venn diagram of overlap between the SPT-Responder signature with Hallmark E2F, Hallmark Androgen Response, and PCa-GI signature. (**D**) Correlation of SPT-Responder

signature and Hallmark E2F (top) and Hallmark Androgen Response (bottom) in SU2C-IDT CRPC cohort (n=266). The Scatter plots show Pearson correlation, the box plots show the groups based on the median of SPT-Responder signature. (**E**) Cutpoint analysis to determine responder and non-responder patients in the discovery SU2C- IDT patient cohort.

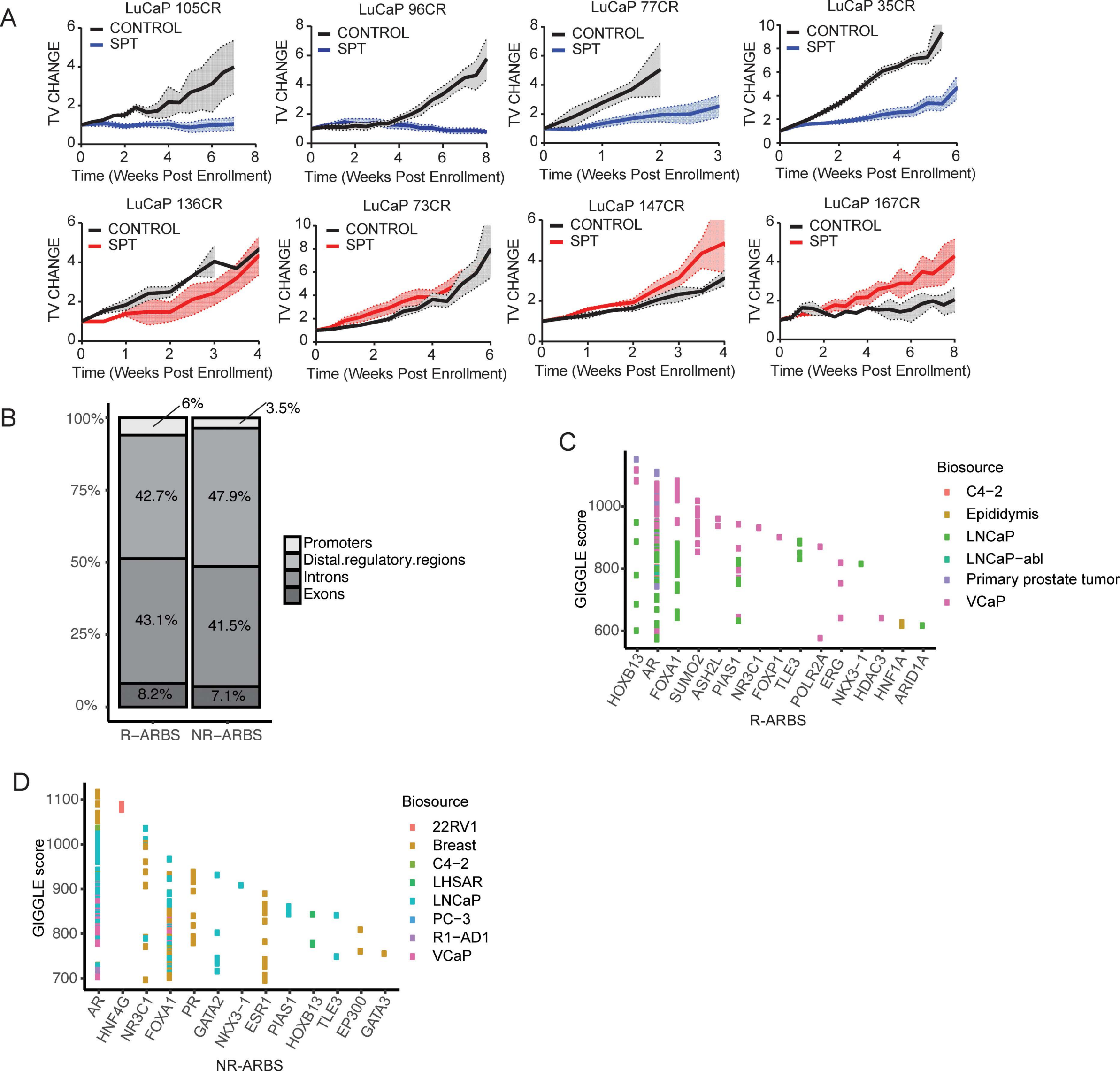
A
LuCaP 105CR
LuCaP 96CR
LuCaP 77CR
LuCaP 35CR
LuCaP 136CR
LuCaP 73CR
LuCaP 147CR
LuCaP 167CR
CONTROL
SPT
TV CHANGE
Time (Weeks Post Enrollment)
B
100%
75%
50%
25%
0%
6%
3.5%
42.7%
47.9%
43.1%
41.5%
8.2%
7.1%
R–ARBS
NR–ARBS
Promoters
Distal.regulatory.regions
Introns
Exons
C
GIGGLE score
HOXB13
AR
FOXA1
SUMO2
ASH2L
PIAS1
NR3C1
FOXP1
TLE3
POLR2A
ERG
NKX3–1
HDAC3
HNF1A
ARID1A
R-ARBS
Biosource
C4−2
Epididymis
LNCaP
LNCaP−abl
Primary prostate tumor
VCaP
D
GIGGLE score
AR
HNF4G
NR3C1
FOXA1
PR
GATA2
NKX3–1
ESR1
PIAS1
HOXB13
TLE3
EP300
GATA3
NR-ARBS
Biosource
22RV1
Breast
C4−2
LHSAR
LNCaP
PC-3
R1-AD1
VCaP

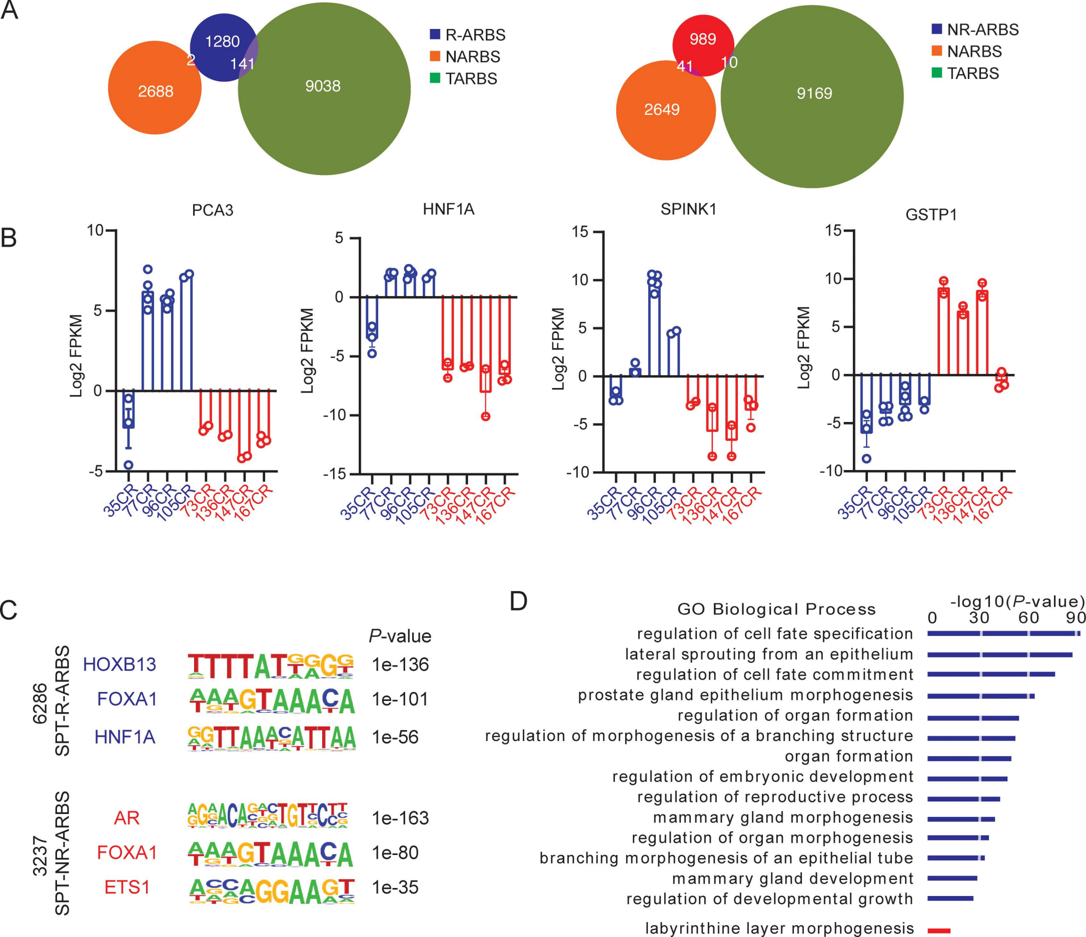
A
R-ARBS
NARBS
TARBS
1280
2
141
2688
9038
NR-ARBS
NARBS
TARBS
989
41
10
2649
9169
B
PCA3
HNF1A
SPINK1
GSTP1
Log2 FPKM
35CR
77CR
96CR
105CR
73CR
136CR
147CR
167CR
C
P-value
6286 SPT-R-ARBS
HOXB13 1e-136
FOXA1 1e-101
HNF1A 1e-56
3237 SPT-NR-ARBS
AR 1e-163
FOXA1 1e-80
ETS1 1e-35
D
GO Biological Process
-log10(P-value)
0
30
60
90
regulation of cell fate specification
lateral sprouting from an epithelium
regulation of cell fate commitment
prostate gland epithelium morphogenesis
regulation of organ formation
regulation of morphogenesis of a branching structure
organ formation
regulation of embryonic development
regulation of reproductive process
mammary gland morphogenesis
regulation of organ morphogenesis
branching morphogenesis of an epithelial tube
mammary gland development
regulation of developmental growth
labyrinthine layer morphogenesis

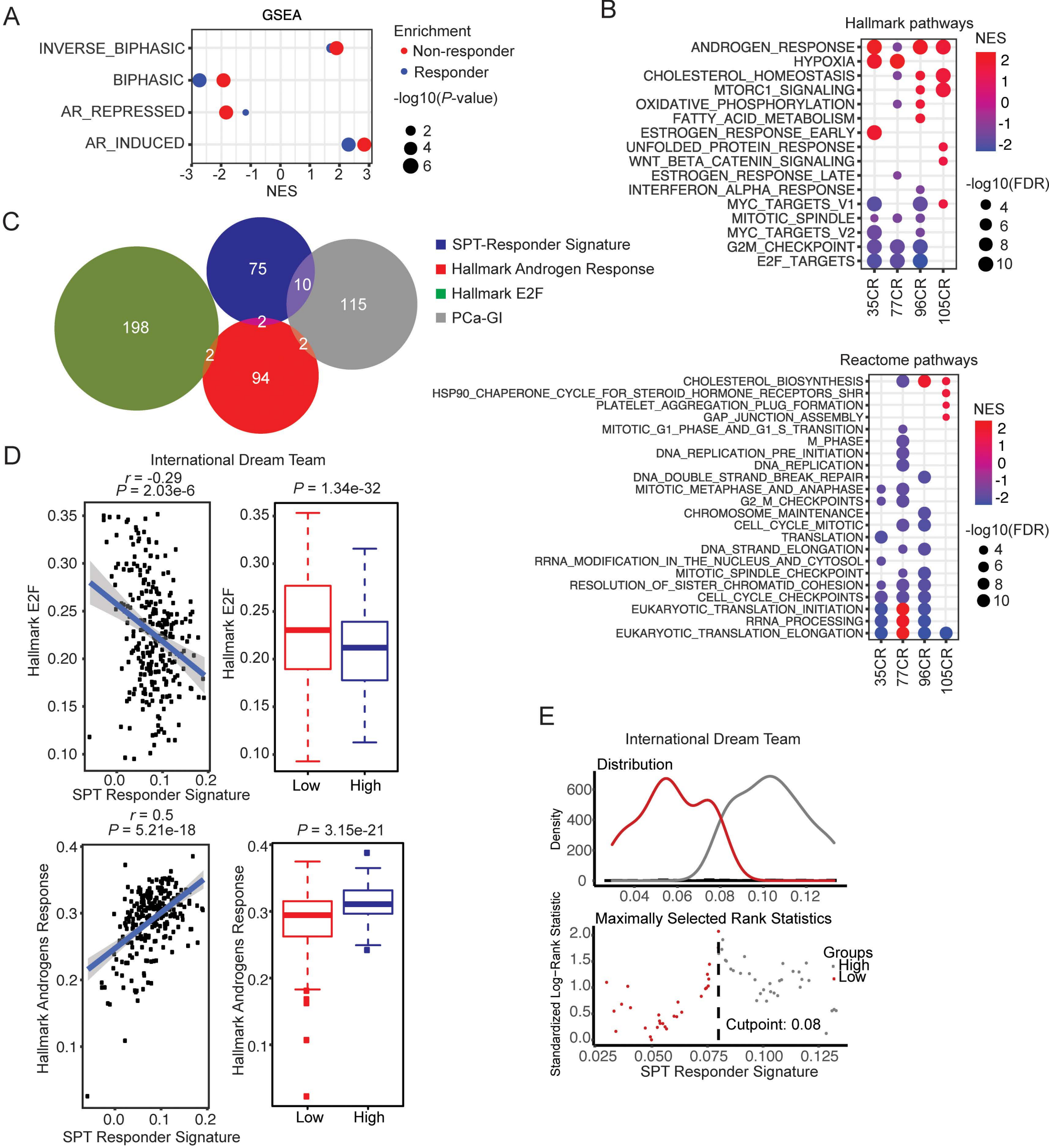

A
GSEA
INVERSE_BIPHASIC
BIPHASIC
AR_REPRESSED
AR_INDUCED
-3 -2 -1 0 1 2 3
NES
Enrichment
Non-responder
Responder
-log10(P-value)
2
4
6
B
Hallmark pathways
ANDROGEN_RESPONSE
HYPOXIA
CHOLESTEROL_HOMEOSTASIS
MTORC1_SIGNALING
OXIDATIVE_PHOSPHORYLATION
FATTY_ACID_METABOLISM
ESTROGEN_RESPONSE_EARLY
UNFOLDED_PROTEIN_RESPONSE
WNT_BETA_CATENIN_SIGNALING
ESTROGEN_RESPONSE_LATE
INTERFERON_ALPHA_RESPONSE
MYC_TARGETS_V1
MITOTIC_SPINDLE
MYC_TARGETS_V2
G2M_CHECKPOINT
E2F_TARGETS
35CR 77CR 96CR 105CR
NES
2 1 0 -1 -2
-log10(FDR)
4 6 8 10
Reactome pathways
CHOLESTEROL_BIOSYNTHESIS
HSP90_CHAPERONE_CYCLE_FOR_STEROID_HORMONE_RECEPTORS_SHR
PLATELET_AGGREGATION_PLUG_FORMATION
GAP_JUNCTION_ASSEMBLY
MITOTIC_G1_PHASE_AND_G1_S_TRANSITION
M_PHASE
DNA_REPLICATION_PRE_INITIATION
DNA_REPLICATION
DNA_DOUBLE_STRAND_BREAK_REPAIR
MITOTIC_METAPHASE_AND_ANAPHASE
G2_M_CHECKPOINTS
CHROMOSOME_MAINTENANCE
CELL_CYCLE_MITOTIC
TRANSLATION
DNA_STRAND_ELONGATION
RRNA_MODIFICATION_IN_THE_NUCLEUS_AND_CYTOSOL
MITOTIC_SPINDLE_CHECKPOINT
RESOLUTION_OF_SISTER_CHROMATID_COHESION
CELL_CYCLE_CHECKPOINTS
EUKARYOTIC_TRANSLATION_INITIATION
RRNA_PROCESSING
EUKARYOTIC_TRANSLATION_ELONGATION
35CR 77CR 96CR 105CR
NES
2 1 0 -1 -2
-log10(FDR)
4 6 8 10
C
75
10
115
198
2
2
2
94
SPT-Responder Signature
Hallmark Androgen Response
Hallmark E2F
PCa-GI
D
International Dream Team
r = -0.29
P = 2.03e-6
Hallmark E2F
0.35 0.30 0.25 0.20 0.15 0.10
0.0 0.1 0.2
SPT Responder Signature
P = 1.34e-32
Hallmark E2F
Low High
r = 0.5
P = 5.21e-18
Hallmark Androgens Response
0.4 0.3 0.2 0.1
0.0 0.1 0.2
SPT Responder Signature
P = 3.15e-21
Hallmark Androgens Response
Low High
E
International Dream Team
Distribution
Density
600 400 200 0
0.04 0.06 0.08 0.10 0.12
Maximally Selected Rank Statistics
Standardized Log-Rank Statistic
2.0 1.5 1.0 0.5 0.0
Groups
High
Low
Cutpoint: 0.08
0.025 0.050 0.075 0.100 0.125
SPT Responder Signature

Figure S3
